# Some dietary factors can modulate the effect of the zinc transporters 8 polymorphism on the risk of metabolic syndrome

**DOI:** 10.1038/s41598-017-01762-9

**Published:** 2017-05-10

**Authors:** Firoozeh Hosseini-Esfahani, Parvin Mirmiran, Gelareh Koochakpoor, Maryam S. Daneshpour, Kamran Guity, Fereidoun Azizi

**Affiliations:** 1 Nutrition and Endocrine Research Centre, Research Institute for Endocrine Sciences, Shahid Beheshti University of Medical Sciences, Tehran, Iran; 2 Faculty of Nutrition Sciences and Food Technology, National Nutrition and Food Technology Research Institute, Shahid Beheshti University of Medical Sciences, Tehran, Iran; 3 Maragheh University of Medical Sciences, Maragheh, Iran; 4 Cellular Molecular and Endocrine Research Centre, Research Institute for Endocrine Sciences, Shahid Beheshti University of Medical Sciences, Tehran, Iran; 5 Endocrine Research Centre, Research Institute for Endocrine Sciences, Shahid Beheshti University of Medical Sciences, Tehran, Iran

## Abstract

There are conflicting data on the impact of zinc transporter 8 (ZNT8) gene variations on the metabolic syndrome (MetS). Hence, the effects of the interaction between rs13266634 and dietary factors on the risk of MetS were investigated in this study. Subjects of this nested case-control study were selected from the participants in Tehran Lipid and Glucose Study. Each of the cases (n = 817) was individually matched with a control. Dietary patterns were determined using factor analysis. The ZNT8 rs13266634 were genotyped by the Tetra-refractory mutation system-polymerase chain reaction analysis. Two dietary patterns were extracted. There were no significant interactions between the ZNT8 SNP and the dietary patterns on the risk of MetS or its components. An interaction was observed between rs13266634 and the omega-3 fatty acid intakes on the risk of MetS in subjects with the CC genotype (P interaction < 0.01). Zinc modified the association of the ZNT8 variant with high fasting blood sugar (P interaction = 0.05) in CC genotype carriers. An interaction was also observed between rs13266634 and salty snacks at the risk of abdominal obesity (P interaction < 0.05). Our findings suggest an interaction between omega-3 fatty acids, zinc, salty snacks and rs13266634, which may affect the risk of MetS or its components.

## Introduction

Zinc transporter 8 (ZNT8), a member of the ZNT family, mainly expressed in pancreatic islet beta cells, is responsible for transporting zinc from the cytoplasm into insulin secretory granules and the subsequent crystallization of insulin^1, 2^.

After understanding the role of zinc in the development of metabolic diseases such as diabetes and metabolic syndrome (MetS)^3^, a common polymorphism in the ZNT8 gene (SL30A8 rs13266634), a C to T variant (arginine to tryptophan in coded protein), has attracted the attention of several genome-wide association studies (GWAS). In some of these studies, this SNP (C allele) was associated with a higher risk of type-II diabetes (T2D), the MetS or its components^4, 5^, a relationship not seen in other studies^6, 7^, which explains only approximately 10% of the overall heritable risk of T2D. This contradiction among the study results and the inability of ZNT8 gene to fully explain the overall heritable risk of T2D^8^ indicates the existence of a complex interaction among rs13266634 and environmental factors e.g. diet and physical activity in relation to the MetS or its components. Based on our knowledge, the effect of the interaction among rs13266634 and dietary factors has been investigated in few studies and the only interactive effects of one or two nutrients (zinc and magnesium) have been examined^9, 10^; hence, the insufficient data available on the effect of the interaction among nutrients and SL30A8 polymorphisms limits the clinical application of these findings. On the other hand, in order to understand the effects of overall diets by obtaining dietary patterns, we investigated the effects of interaction of ZNT8 rs13266634 with dietary patterns, macronutrients, and some minerals or food groups at the risk of MetS and related components in this nested case-control study.

## Materials and Methods

### Study population

Subjects of this nested case-control study were selected among participants of the Tehran Lipid and Glucose Study (TLGS), a large-scale community-based prospective study being performed on the sample of residents of District 13 of Tehran, the capital of Iran. The first phase of the TLGS was conducted from 1999 to 2001 on 15,005 subjects, aged ≥3 years, and follow-up examinations have been conducted every 3 years (2002–2005, 2006–2008, 2008–2011, and 2011–2014) to identify newly developed diseases. Details of this ongoing cohort study have been published elsewhere^11, 12^.

Of 11,001 and 9,807 individuals, aged ≥18 years, who participated in the baseline and second follow-up surveys, respectively, as many as 5,280 were excluded for developing the MetS at either baseline or for the second follow-up survey. In the current study, among the participants, who developed the MetS in the third (n = 918), fourth (n = 827) or fifth (n = 1050) phases, 1,198 cases were randomly selected with respect to the sample size. After excluding individuals with a history of cardiovascular events, weight loss or gain of more than 5 kg in the last 6 months, pregnancy, and lactation, or those taking any anticoagulant, steroid, any medicine against cardiovascular disease or hormonal disorder, 1,158 cases were included in the study. Each case was individually paired randomly with a control by age (±5 years) and sex among those who had not developed ≥1 MetS components at the time when the corresponding case developed the MetS. After excluding cases or controls that lack DNA purification in the range of 1.7 < A260/A280 < 2, and whose reported energy intake divided by the predicted energy intake did not qualify for the ±3 SD range, the final data of 1,634 subjects with the MetS and matched controls (817 pairs) remained for analysis.

Informed written consents were obtained from all the participants. The study protocol was approved by the ethics committee of the Research Institute for Endocrine Sciences, Shahid Beheshti University of Medical Sciences in Tehran and the medical research ethics committee of Jundishapur University of Medical Sciences, Ahvaz, Iran. All the methods were performed in accordance with their relevant guidelines and regulations.

### Measurements

The dietary intake was assessed with the use of a valid and reliable 168-item semi-quantitative food frequency questionnaire (FFQ) to obtain the usual food intake of the individuals during the 12 months before the examination. The consumption frequency of each food item on a daily, weekly or monthly basis was converted to daily intakes and portion sizes were then converted to grams using household measures. Because the Iranian food composition table (FCT) is incomplete, we used the United States Department of Agriculture (USDA) FCT to analyse food and beverages^13^. However, the Iranian FCT was used for some traditional food and beverages, not listed in the USDA FCT^14^. Based on the macronutrient composition and using current literature, 25 food groups were categorized^15–17^.

The anthropometric assessment was done using a standardized process. Weight was measured to the nearest 100 g, using digital scales while the subjects were minimally clothed and not wearing shoes. Subjects’ height was measured without shoes to the nearest 0.5 cm with a measuring tape in a standing position and with shoulders resting at a standard alignment. Waist circumference (WC) was measured to the nearest 0.1 cm, at the umbilical level over light clothing, using an upstretched meter tape without any pressure to the body surface. Blood pressure (BP) was measured on the right arm in a sitting position twice with a 15-minute gap, and finally, the mean of the two measurements was reported as the subject’s BP.

The physical activity level was assessed using the Persian-translated modifiable activity questionnaire (MAQ) with high reliability and relatively moderate validity. The frequency and time spent on activities with light, moderate, hard and very hard levels of intensity, according to the list of daily common activities over the past year were obtained, and the data was transformed into metabolic equivalent hours per week (METs/h/wk)^18–20^.

Fasting blood samples were taken after 10–12 hours of overnight fasting. Fasting plasma glucose (FPG) and triglycerides (TG) were measured using the enzymatic colorimetric method and high-density lipoprotein cholesterol (HDL-C) was measured after the precipitation of apolipoprotein β with phosphotungstic acid. These analyses were performed using the Pars Azmoon kits (Tehran, Iran) and a Selectra-2 auto-analyser (Vital Scientific, Spankeren, Netherlands).

### Genotyping

The ZNT8 genes contain a common variant - rs13266634 - the association of which with the T2D and the MetS has received much attention. Hence, the genomic DNA was extracted from the peripheral blood using standard salting-out method^21^. The selected polymorphism (rs13266634) was studied using the tetra-primer refractory mutation system-polymerase chain reaction (T-ARMS-PCR) method. The primers were determined through the national centre for biotechnology information (NCBI) site^22^. Our T-ARMS assay with different inner allele specific primers produced allele-specific PCR products; forward: CTT CTT TAT CAA CAG CAG CCC GCC, and reverse: TCT CCG AAC CAC TAG GCT GTA CCA. Two outer primers produced a PCR product, which is to be used as an internal control for reaction; forward: GAA GTT GGA GTC AGA GCA GTC GCC, and reverse: ATC TCA GTG CCT CTT CCT TCA TGG TGA. For the SNP mentioned, the PCR reaction was optimized at a 12.5 µl total volume containing 1 µl DNA template, 6.25 µl master mix containing MgCl_2_, Smart Taq polymerase (CinnaGene Co, Iran), BSA 0.1% (TaKaRa, Japan), 1.8 µl total primers (containing 0.6 µl outers and 1.2 µl inners), 0.05 µl Mgcl_2_, and 3.4 µl water.

The PCR amplification was carried out with an initial denaturation at 94 °C for 6 minutes, denaturation at 95 °C for 30 seconds (35 cycles), 35 seconds of annealing at 64.8 °C (35 cycles), 55 seconds of extension at 72 °C, and an additional 10 minutes of extension at 72 °C at the end of the final cycle. The PCR products were resolved by electrophoresis on a 1.8% agarose gel, a procedure that rendered three types of bands in heterozygotes (391, 334, and 104 bp) and two types of bands in homozygotes (mutant allele carrier resulting in 391 and 104 bp, wild carrier allele resulting in 391 and 334 bp). A 391-bp band was always obtained as the control for the success of the amplification. In order to validate the accuracy of genotype scoring by T-ARMS-PCR, the three (391, 334 and 104 bp) fragments were directly sequenced (for 10% of the samples). The genotyping result was rechecked using Illumina chip as the whole genome sequencing, the other SNP rs16889462 immediately adjacent to, and in the same codon with rs13266634. The old result was confirmed with the chip. Also, the allele and genotype frequency of other marker (rs16889462) was A: 0.54, G: 99.46, AA: 0.01, AG: 0.86, GG: 99.13%. Hence, the other marker didn’t have any effect of genotyping with T-ARMS-PCR.

### Definitions

The MetS were defined with respect to the modified definition of the National Cholesterol Education Program/Adult Treatment panel III (ATP III)^23^, as having three or more of the following criteria: (1) abdominal obesity (WC ≥ 95 cm for both genders) according to the newly-introduced cut-off points for Iranian adults^24^, (2) high TG (≥150 mg/dL) or drug treatment, (3) low HDL-C (<40 mg/dL in men or <50 mg/dL in women) or drug treatment, (4) high blood pressure (SBP/DBP ≥ 130/85 mmHg) or antihypertensive drug treatment, and 5) high FPG (≥110 mg/dL) or drug treatment for elevated glucose.

### Statistical analysis

For the descriptive analysis, a comparison of the qualitative and quantitative variables among cases and controls was done using the student T and Chi-square test statistics, respectively. The TG concentration (a non-symmetric quantitative variable) was log-transformed before the statistical analysis. The genotype and allele frequencies for the analysed polymorphism were obtained using the Power-Marker software. Pearson’s Chi-square statistic was used to calculate the Hardy-Weinberg equilibrium.

The dietary patterns were identified using factor analysis with varimax rotation, based on 25 food groups. The patterns were then extracted based on the eigenvalues (>1), scree plot, factor interpretability, and the variance explained (>5%).

Conditional logistic regression was used to estimate the interactions of the SNP with percentiles of dietary factors in relation to the MetS after applying an adjustment for the baseline BMI. Two likelihood scores were obtained, performing this statistical analysis, with and without the interaction terms. The P value for interaction was determined by performing the likelihood ratio test. The conditional logistic regression was also used to generate the odds ratios (ORs) for the MetS among individuals in genotype groups (CC/CT + TT) across percentiles of dietary pattern scores (Q1–Q4), food groups, and nutrient intakes (T1–T3). The highest quartile of dietary factors and the CT + TT genotype group were examined as the reference group.

Unconditional logistic regression was performed to estimate the interactions of the SL30A8 SNP with percentiles of dietary pattern scores, food groups, and nutrients intake in relation to the MetS components. All the ORs were adjusted for variables proven to be associated with the MetS components, including age, gender, educational level, smoking status, physical activity, and energy intake. In order to determine the P value for trend across the percentile of dietary factors, logistic regression was applied using the median of each percentile of dietary factors as a continuous variable. To show the robustness of the results and considering sampling uncertainty, we applied bootstrapping method and repeated the conditional and unconditional logistic regression models on 100 resamples, drawn randomly with replacement^25^. The data were analysed using STATA (Statistics/Data analysis 12.0) or SPSS (Statistical Package for Social Sciences, Version 16.0; SPSS, Chicago, IL).

## Results

### Study population

The general characteristics of participants by cases and controls are shown in Table 1. There were no significant differences between the two groups in physical activity, years of education, smoking, or daily energy intake. However, the values of the MetS components significantly differed among cases and controls at the beginning of the study. The genotype frequency was in Hardy-Weinberg equilibrium (P = 0.74). No significant differences were observed in the frequencies of the genotypes or alleles between the two groups (Table 1).

**Table 1:** Characteristics of the study population in subjects with MetScases and controls.

	Without MetS (n = 817)		With MetS (n = 817)	
		SD		SD
Baseline Age (y)	42.94	12	43.2	11
Men	44.3	12	43.6	12
Women	41.7	11	42.8	11
Current smokers (%)	21.8		19.6	
physical activity (MET.h/w)	7.44	12	7.34	13
Education level ≥14 years (%)	11.7		9.5	
Baseline BMI^a^ (Kg/m^2^)	24.9	4	28.5*	4
Obesity (%)^b^	15.8		47.6*	
Baseline WC (cm)	83.1	10	93.2*	11
Abdominal obesity (%)^c^	54.3		90.7*	
Baseline systolic BP (mmHg)	112.3	15	121.79*	17
Baseline diastolic BP (mmHg)	73.8	9	79.5*	10
Elevated BP (%)^d^	23.5		58.9*	
Baseline HDL-C (mg/dl)	44.8	9	39.2*	10
Low HDL-C (%)^e^	28.6		82.8*	
Baseline TG (mg/dl)	102.5	40	164.0*	71
High TG (%)^f^	14.0		68.4*	
Baseline FBG (mg/dl)	86.5	11	109.74*	10
High FBG (%)^g^	21.9		79.1*	
Energy intake (Kcal/day)	2420.4	1062	2409.1	879
Carbohydrate (% of energy)	59.0	8	59.4	9
Total fat (% of energy)	30.13	8	29.87	7.2
Saturated fat (% of energy)	10.13	3	9.9	3
MUFA (% of energy)	10.0	3	10.0	2
PUFA (% of energy)	6.1	2	6.1	2
Allele frequency rs13266634 n (%)				
T	400(24)		370(22)	
C	1232(75)		1264(77)	
Genotype frequency rs13266634 n (%)				
TT	52(6.4)		39(4.8)	
TC	296(36.2)		292(35.7)	
CC	496(57.4)		486(59.5)	

BMI: body mass index. WC, waist circumference. BP. blood pressure. HDL-C. high density lipoprotein cholesterol. TG: triacylglycerol. FBG, fasting blood glucose, MUFA, Mono-unsaturated fatty acids. PUFA, Poly-unsaturated fatty acids *P < 0.05; ^†^values are mean unless otherwise listed. ^a^Baseline BMI at the first or second phase of TLGS. ^b^BMI ≥ 30 kg/m^2^, ^c^WC ≥ 95 cm for both genders. ^d^BP ≥ 130/85 mmHg, ^e^HDL-C < 40 mg/dl in men and < 50 mg/d in women; ^f^TG ≥ 150 mg/dl, ^g^FBG ≥ 110 mg/dl.

Two major dietary patterns were identified - the healthy dietary pattern was loaded heavily on vegetables, legumes, low-fat dairy, whole grains, fruit juices, liquid oils, and fruits, whereas the western dietary pattern consisted of high intakes of soft drinks, fast foods, sweets, sugar-based food, solid oils, red meat, salty snacks, refined grains, high-fat dairy, tea, coffee, eggs, and other poultry products (Table 2).

**Table 2:** Factor loadings for the two dietary patterns identified in study participants.

Food groups	Dietary patterns^a,b^	
	Western	Healthy
Sweets and Sugar	0.55	
Soft drinks	0.49	
Red meats	0.48	
Solid oils	0.46	
Refined grains	0.45	
Fast foods	0.45	
Salty snacks	0.40	0.21
Eggs	0.39	0.26
Tea and coffee	0.36	
High fat dairy	0.36	0.29
Poultry	0.27	
Non Starchy vegetables		0.63
Starchy vegetables		0.59
Legumes		0.58
Fruit juice		0.47
Low fat dairy		0.45
Whole grains		0.44
Liquid oils	0.258	0.38
Fruits		0.23
Fish		
Nuts and seeds		
Variance (%)	10.47	10.69

^a^Values are factor loadings of dietary patterns measured by factor analysis. Factor loadings below ±0.2 are not shown in the table for simplicity. ^b^Eigenvalues > 1, Kaiser-Meyer-Olkin (KMO): 0.74.

### Interactions among SNP and dietary factors in relation to the MetS

After applying the adjustment for the baseline BMI, there was no significant interaction among the ZNT8 SNP and the dietary patterns in relation to the MetS. Among the nutrients examined in this study, only omega-3 fatty acid intakes could modulate the association of genotype groups of rs13266634 with the MetS (P interaction = 0.009); carriers of CC genotype benefited more from the omega-3 fatty acid intakes than carriers of the CT + TT genotypes; CC genotype carriers, who consumed more omega-3 fatty acids, had a lower risk of developing the MetS, whereas no such relationship was observed in the carriers of CT + TT genotypes (Table 3).

**Table 3:** Adjusted ORs (95% CI) for MetS according to quartiles of dietary pattern scores^a^.

		Q1	Q2	Q3	Q4	P for trend	P for interaction
**Healthy dietary pattern**							
CC		**1.26**	**1.35**	**1.18**	**1.39**	0.53	0.21
	CI	(0.67–2.08)	(0.84–2.15)	(0.71–1.94)	(0.86–2.26)		
	CI^b^	(0.77–2.08)	(0.90–2.02)	(0.70–1.97)	(0.83–2.30)		
CT + TT		**1.14**	**0.90**	**1.26**	**1**	0.8	
	CI	(0.67–1.94)	(0.52–1.53)	(0.75–2.12)			
	CI^b^	(0.66–1.96)	(0.53–1.51)	(0.73–2.17)			
**Western dietary pattern**							0.74
CC		**1.50**	**1.44**	**1.60**	**1.52**	0.42	
	CI	(0.89–2.53)	(0.88–2.36)	(0.98–2.61)	(0.92–2.50)		
	CI^b^	(0.94–2.40)	(0.89–2.32)	(0.99–2.60)	(0.89–2.59)		
		**1.45**	**1.04**	**0.97**	**1**	0.25	
CT + TT	CI	(0.84–2.49)	(0.92–2.70)	(0.55–1.68)			
	CI^b^	(0.80–2.63)	(0.94–2.64)	(0.55–1.68)			
**Omega-3 Fatty acids**							**0.009**
CC		**2.86**	**1.38**	**1.05**	**0.50**	**0.001**	
	CI	(1.35–3.52)	(0.84–2.28)	(0.65–1.69)	(0.28–0.86)		
	CI^b^	(1.37–3.48)	(0.90–2.02)	(0.61–1.75)	(0.27–0.88)		
CT + TT		**1.07**	**1.46**	**1.13**	**1**	0.65	
	CI	(0.64–1.78)	(0.84–2.21)	(0.66–1.93)			
	CI^b^	(0.70–1.62)	(0.80–2.33)	(0.71–1.87)			

OR: Odds Ratio, Q: Quartiles of dietary pattern scores or omega 3 fatty acid (Q1 < 0.33, Q2:0.33–0.45, Q3:0.45–0.58 and Q4 > 0.58% of energy). ^a^ORs (95% CI) were calculated by using conditional logistic regression model, adjusted for baseline BMI and the interaction term (SNP × dietary pattern scores). Participants were classified (8 groups) according to quartiles of dietary pattern scores and rs13266634 genotypes. The highest quartile of dietary pattern scores and homozygote genotype of major allele were used as the reference group. ^b^CI after Bootstrap analysis.

### Interactions of SNP and dietary factors in relation to the MetS components

**Figure 1:**
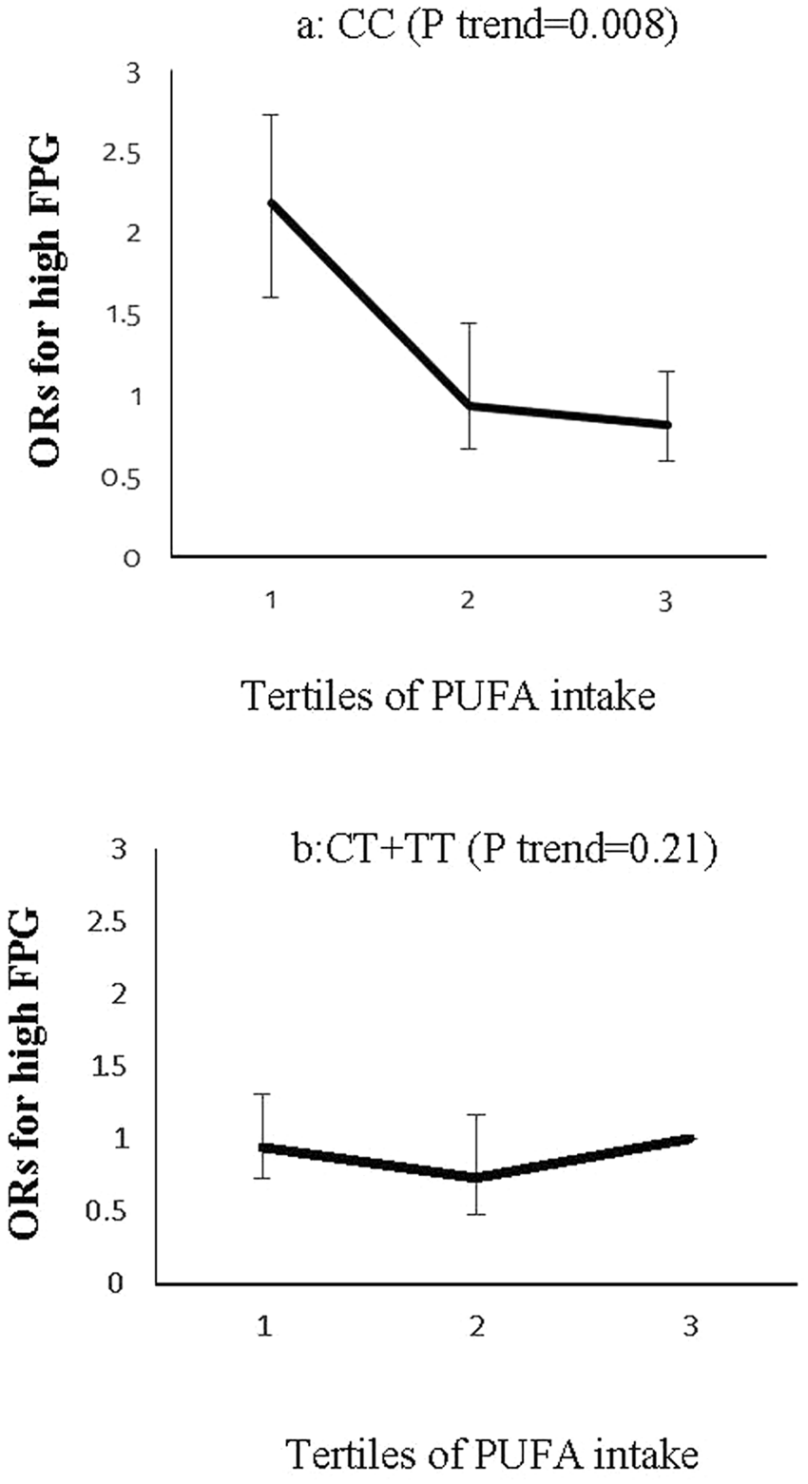
Adjusted ORs (95% CI) for high FPG (Fasting Plasma Glucose) across tertiles of polyunsaturated fatty acids (PUFA) by the ZNT8 genotypes (P interaction = 0.03); CIs are shown after the bootstrap analysis (T1 < 4.93, T2:4.93–6.58., T3 > 6.58% of energy), in the CC genotype carriers, the risk of high FPG decreased across tertiles of PUFA intake (P trend = 0.008) (**a**), an association, however, not significant in the CT + TT genotype groups (**b**).

**Figure 2:**
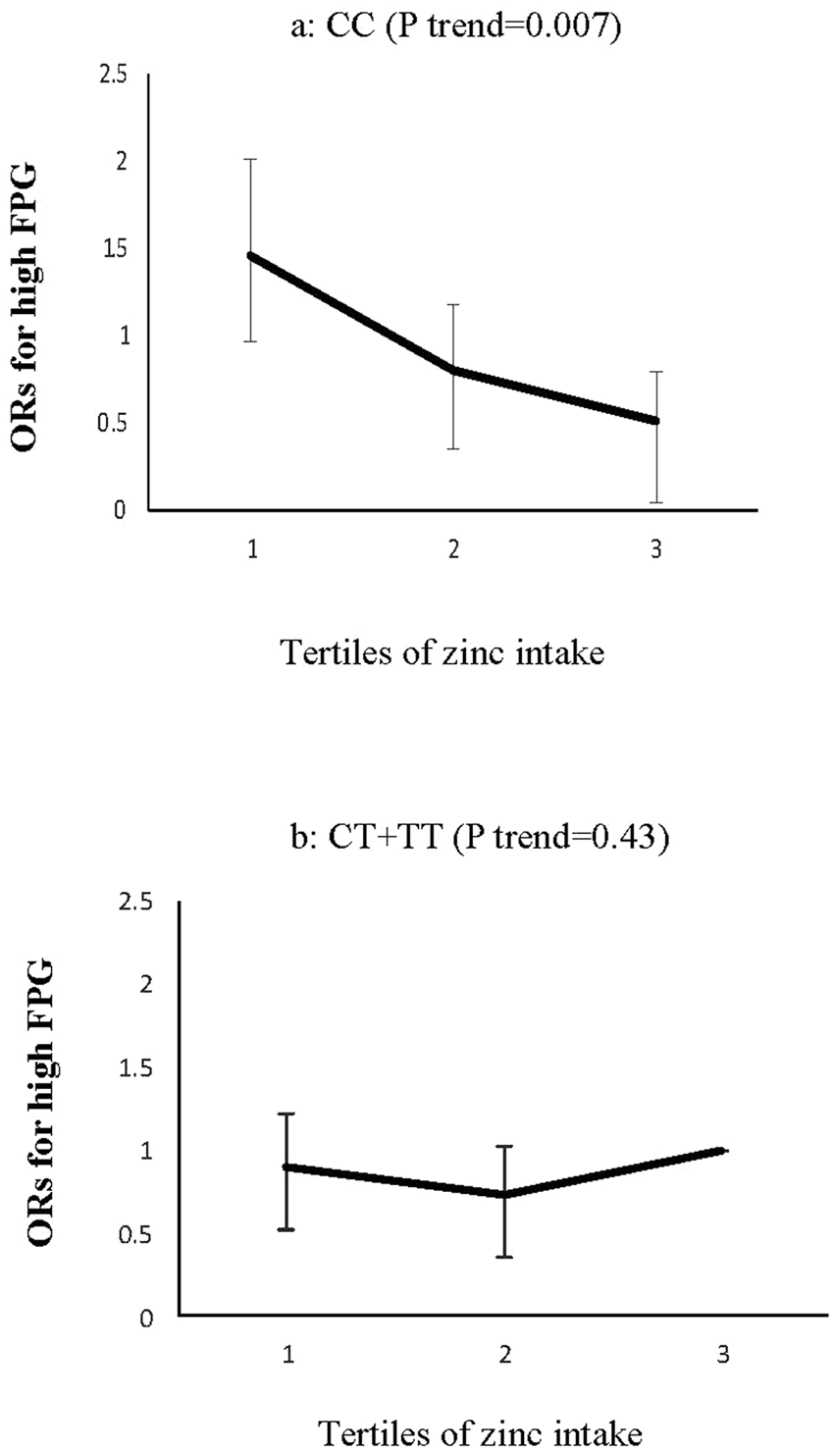
Adjusted ORs for high FPG (Fasting Plasma Glucose) across tertiles of zinc intake by the ZNT8 genotypes (P interaction = 0.05); CIs are shown after the bootstrap analysis (T1 < 10.52 mg, T2:10.52–14.24 mg, T3 > 14.24 mg), in the CC genotype carriers, the risk of high FPG decreased across tertiles of zinc intake (P trend = 0.007) (**a**), an association, however, not significant in the CT + TT genotype groups (**b**).

There was a significant interaction among the ZNT8 rs13266634 and the dietary polyunsaturated fatty acids (P interaction = 0.03) and zinc (P interaction = 0.05) at the risk of high FPG; so that the risk of high FPG decreased across tertiles of polyunsaturated fatty acid (P trend = 0.008) and zinc intake (P trend = 0.007) in the CC genotype carriers, an association that was not significant in the CT + TT genotype groups (Figs 1 and 2).

**Figure 3:**
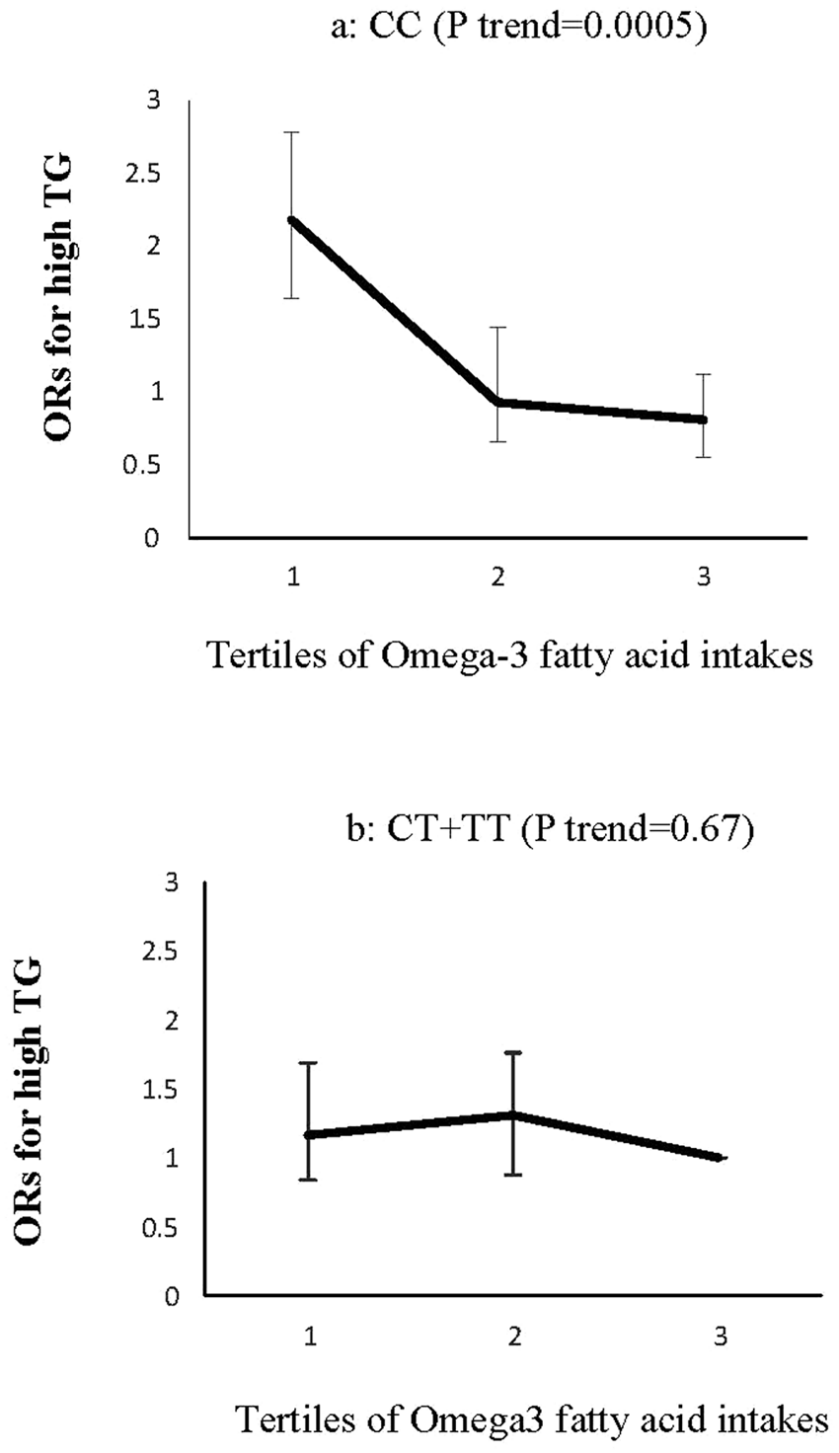
Adjusted ORs for high triglycerides (TG) across tertiles of omega-3 fatty acid intakes by the ZNT8 genotypes (P interaction = 0.05); CIs are shown after the bootstrap analysis (T1 < 0.38, T2:0.38–0.54., T3 > 0.54% of energy). The risk of high TG decreased across tertiles of omega-3 fatty acid intakes in CC genotype carriers (**a**), a trend not observed in the CT + TT genotype groups (**b**).

**Figure 4:**
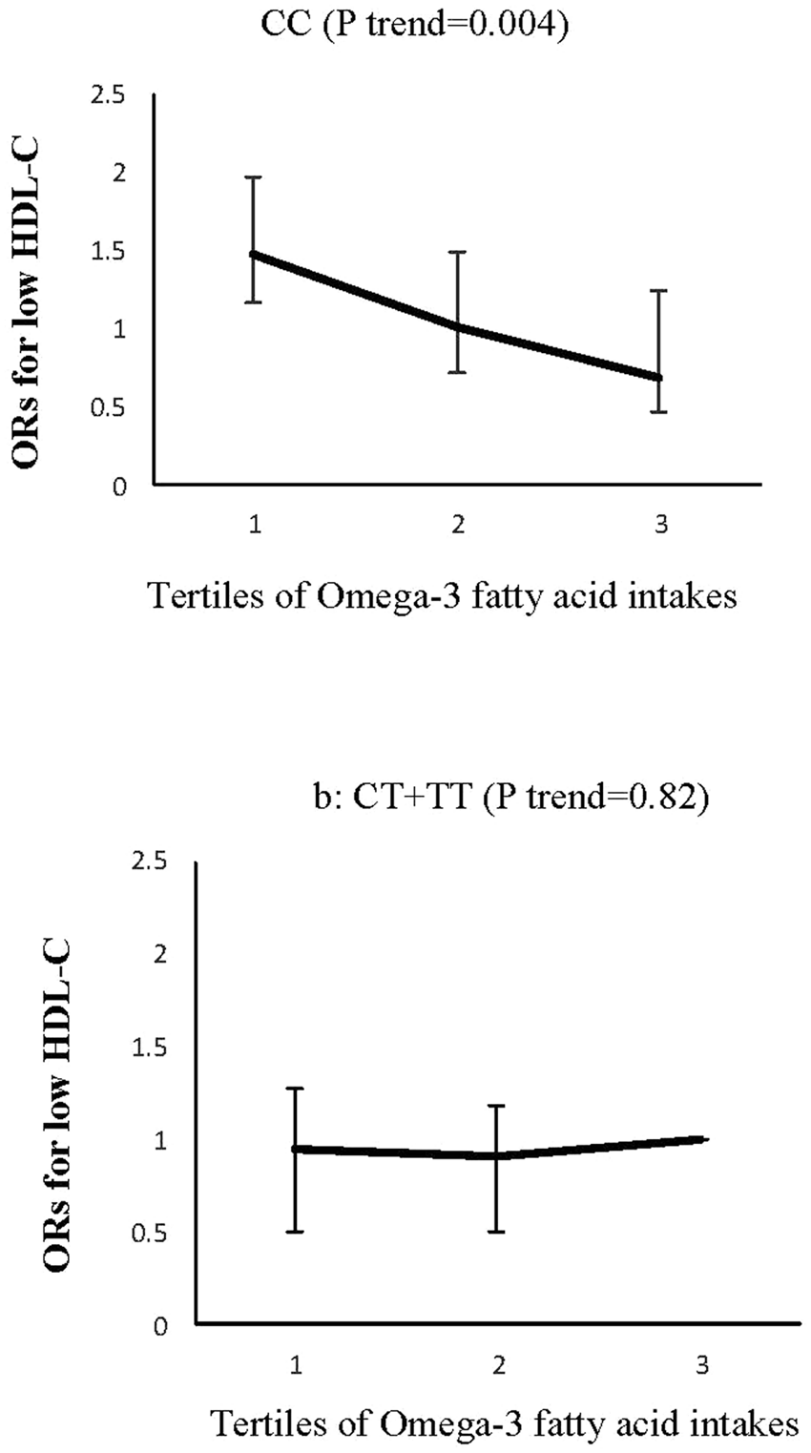
Adjusted ORs for low HDL-C to tertiles of omega-3 fatty acid intake by the ZNT8 genotypes (P interaction = 0.02); CIs are shown after the bootstrap analysis, (T1 < 0.38, T2:0.38–0.54., T3 > 0.54%of energy). The risk of low HDL-C decreased across tertiles of omega-3 fatty acid intakes in CC genotype carriers (**a**), a trend not observed in CT + TT genotype groups (**b**).

A significant interaction was also observed among rs13266634 and omega-3 fatty acids in relation to the risk of high TG (P interaction = 0.001), and low HDL-C (P interaction = 0.03). The risk of dyslipidemia decreased across the tertiles of omega-3 fatty acid intakes in the CC genotype carriers, a trend not observed in the CT + TT genotype groups (Figs 3 and 4).

**Figure 5:**
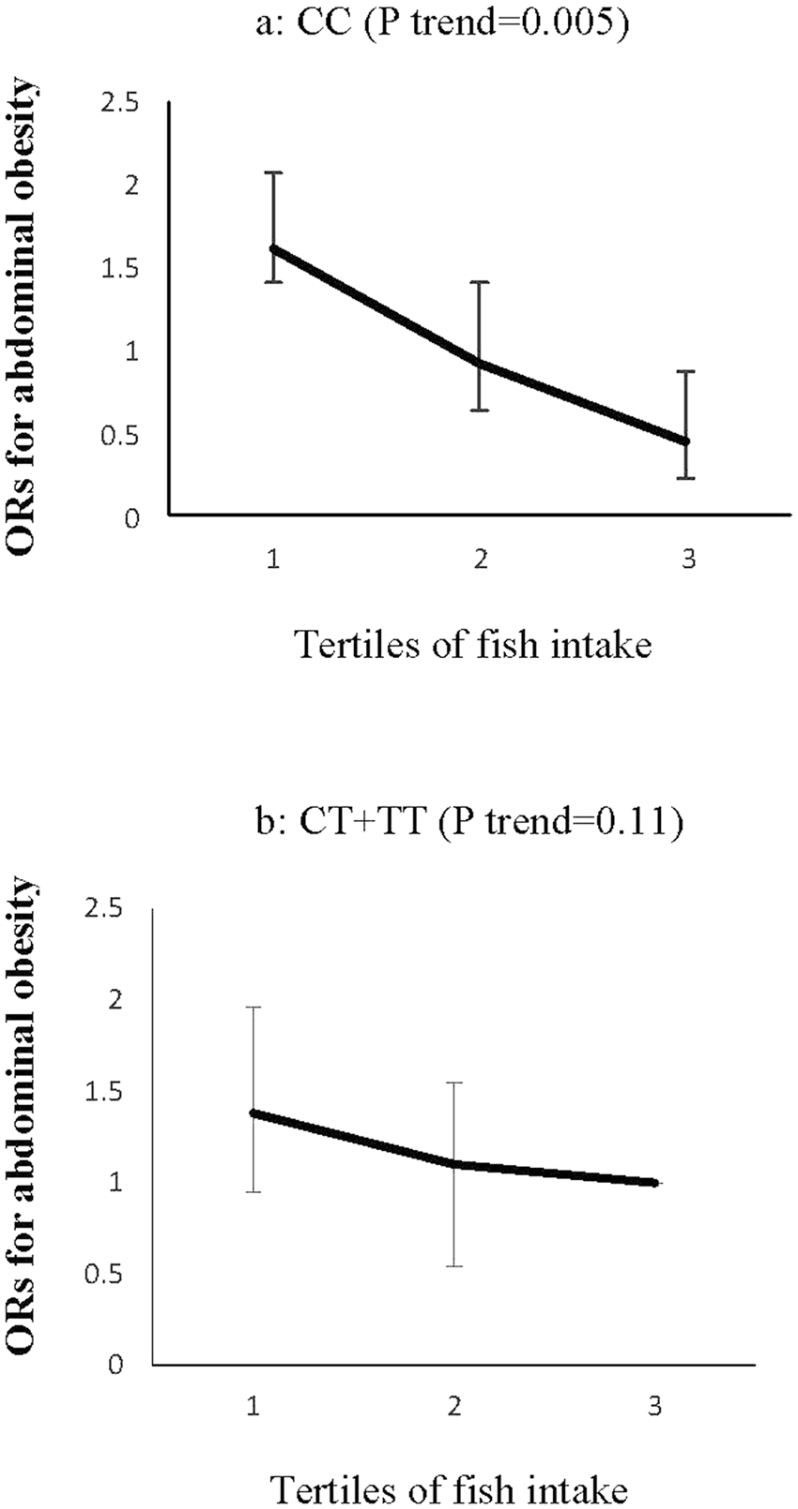
Adjusted ORs for abdominal obesity across tertiles of fish intake (variety of fish and tuna) by the ZNT8 genotypes (P interaction = 0.04); CIs are shown after the bootstrap analysis, (T1 < 4.23, T2:4.23–10.86, T3 > 10.86 gr/day). The ORs for abdominal obesity decreased significantly with increased consumption of fish by CC genotype carriers (P trend = 0.005) (**a**), although consumption of this food group had no significant effect on the ORs for abdominal obesity in the CT + TT genotype groups (P trend = 0.11) (**b**).

**Figure 6:**
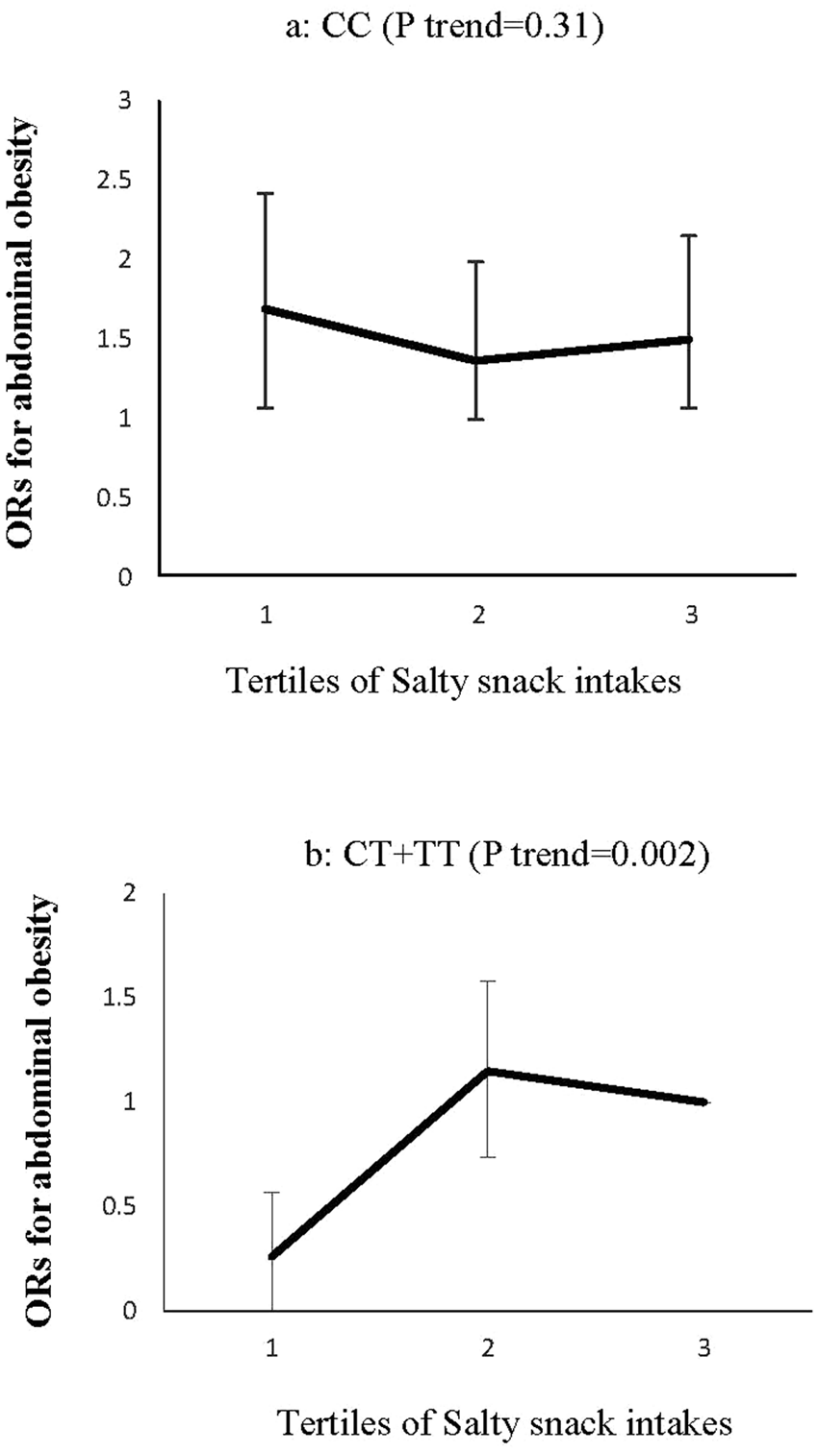
Adjusted ORs for abdominal obesity across tertiles of salty snack intakes **(**Crackers, pretzels chips, pickles and salted vegetables) by ZNT8 genotypes (P interaction = 0.02); CIs are shown after the bootstrap analysis, (T1 < 2.83, T2:2.83–10.70, T3 > 10.70 gr/day). In the CT + TT genotype groups, the risk of abdominal obesity increased across tertiles of salty snack intakes (P trend = 0.02) (**a**), an association not significant in the CC homozygote carriers (P trend = 0.31) (**b**).

Among food groups, fish (P interaction = 0.04) and a salty snack (P interaction = 0.02) intakes changed the association of the ZNT8 variant with abdominal obesity. The ORs for abdominal obesity decreased significantly with increased consumption of fish by the CC genotype carriers (P trend = 0.005), although consumption of this food group had no significant effect on the ORs for abdominal obesity in the CT + TT genotype groups (P trend = 0.11) (Fig. 5). In the CT + TT genotype groups, the risk of abdominal obesity increased across tertiles of salty snack intakes (P trend = 0.02), an association not significant in the CC homozygote carriers (P trend = 0.31) (Fig. 6).

No significant interactions among the ZNT8 rs13266634 and other dietary factors, in relation to the MetS components, were observed.

## Discussion

The present study provides information about the effect of the interaction of dietary factors and a genetic polymorphism of ZNT8 (rs13266634), in association with the risk of developing the MetS and its components.

Neither of the two dietary patterns identified in this study modulated the relationship of the ZNT8 polymorphism with the MetS or its components. No study has yet investigated the interactive effect of the dietary patterns of the association among the ZNT8 variant and the MetS. However, previous studies have revealed that dietary patterns can alter the effects of other polymorphisms on the MetS^26^. The dietary patterns represent combinations of food groups describing as much variance in the original dietary variables but not necessarily those combinations of foods that show the strongest interaction.

Despite the ineffectiveness of dietary patterns identified in this study, omega-3 fatty acid consumption could alter the association among the ZNT8 polymorphism and the MetS. There was a significant interaction among the sources of omega-3 fatty acid consumption and the ZNT8 polymorphism, in relation to the MetS, dyslipidaemia, and abdominal obesity; such that the participants with the CC genotype exhibited a lower risk of developing the MetS, dyslipidaemia and abdominal obesity with the increase in omega-3 fatty acid consumption. Hence, compared with the CT + TT genotype carrier, intake of one percent of the energy requirement through the consumption of omega-3 fatty acids in the CC genotype carriers reduced the risk of high TG and low HDL-C by 19% and 32%, respectively. Our findings support previous studies that proves that the consumption of omega-3 fatty acids can be effective in the treatment and prevention of the development of MetS and its components. Ebbesson and *et al*. showed that high intake of omega-3 fatty acids by Eskimos led to the development of the MetS and its components^27^.

In this study, the omega-3 fatty acids have been introduced as the strongest modulators of the ZNT8 variant’s effect on the MetS, however, both the healthy and western dietary patterns extracted in this study, had low intakes of the most common sources of omega-3 fatty acids - fish and nuts (factor loadings <0.2). Hence, it is not surprising that there was no significant interaction between the ZNT8 polymorphism and these dietary patterns, in relation to the MetS.

In our study, with higher intakes of omega-3 fatty acids in the CC genotype carriers, the OR for low HDL-C decreased. Blood levels of free fatty acids reduced after the consumption of omega-3 fatty acids^28^, hence, transferring cholesteryl ester (CE) from HDL-C to LDL-C and VLDL (very low-density lipoprotein cholesterol) via Cholesteryl ester transfer protein (CETP) decreased, which resulted in an increase in the HDL-C serum^29^.

According to our results, consumption of fish containing omega-3 fatty acids by the CC genotype carrier is associated with the decreased odds of abdominal obesity. There are several possible explanations for this association. First, compared with saturated fatty acids, polyunsaturated fatty acids are stored less and consumed more for energy supply^30^. Secondly, consumption of unsaturated fatty acids are effective in reducing the sense of hunger^31^ and finally, lean body mass increased with higher intake of polyunsaturated fatty acids^32^.

Based on the results of our study, consumption of polyunsaturated fatty acids and zinc reduces the FPG level in the CC genotype group significantly. It seems that the consumption of PUFA by reducing the level of inflammation and leptin^33^, the consumption of zinc by improving secretion and storage of insulin^34^, the amplification of insulin signalling pathways^35^, and preventing pancreatic β cells death^36^ inhibition of gluconeogenesis^37^ reduce the risk of high FPG.

Besides, our findings suggest that associations among unsaturated fatty acids and zinc with the MetS and its components are not uniform in genotype groups of rs13266634 and that they represent an interaction among rs13266634 and polyunsaturated fatty acids and zinc intakes. These findings support previous studies that reported interactions among polyunsaturated fatty acids and genetic variants in association with intermediate phenotypes (plasma lipid concentrations, glucose, and anthropometric measurements) or disease phenotypes (cardiovascular diseases and MetS)^38^. The unsaturated fatty acids can alter the expression of genes affecting the MetS or its components by binding to a transcription factor gene expression such as PPAR-α^39^. In addition, three CpG sites located nearby rs13266634. The DNA methylation at these CpG sites by nutrients can lead to a decrease in the ZNT8 mRNA expression levels. On the other hand, the results of this study also propose that unsaturated fatty acids can change the occurrence of the phenotype through alterations in the ZNT8 gene expression or via epigenetic modification. The moderating effect of zinc on the ZNT8 polymorphisms was also reported in the Shan *et al*. study^40^, a cross-sectional study, in which the inverse association between plasma zinc and T2D risk was weaker in the CC and CT genotype carriers than in the TT genotype carriers. However, in our study, the risk of high FPG decreased by increasing zinc intake only in the CC genotype group. But this effect was not observed in CT + TT genotype group. Differences in the study design and method used to assess the zinc status or grouping genotypes can justify this controversy. Improvement of ZNT8 gene expression, insulin secretion and glucose homeostasis by increasing zinc intake are the proposed mechanisms for this interaction^41^. There is no doubt that further studies are required to prove these hypotheses and to learn more about the molecular mechanisms of the interaction among ZNT8 genes and nutrients.

In this study, by increasing salty snack intakes in the CT + TT genotypes, the risk of abdominal obesity significantly increased, a finding that is consistent with the results of the Ma *et al*. study^42^ that proves that eating salt increased the chance of obesity, independent of energy intake. Several mechanisms have been proposed for this association, including increased water consumption, a consequent increase in the volume of extracellular water, and changes in fat metabolism following salt intake. Other studies also indicated that the body’s response to salt intake is dependent on the subject’s genetic structure^43^. Though only salty snack intakes were evaluated and salt intake from other sources, including cheese and bread, have not been evaluated in our study, the results suggest that the CC genotype carriers are resistant to salt intake. The findings need further studies to confirm.

The prospective design with a long-term follow-up, a large number of cases, matched individually by age and sex, and extensive adjustment for potential confounders are the strengths of our study. However, it does have its limitations that need to be addressed. The participants were largely homogeneous as the study was performed only on residents of District 13 in Tehran. Besides, as the insulin sensitivity was not measured, we were unable to detect the interaction among the dietary factors and the ZNT8 SNP, in relation to it.

## Conclusions

Results of this study have demonstrated significant interaction among omega-3 fatty acids, zinc, salty snack intakes, and the ZNT8 variant, in relation to the MetS and its components. Compared with the CT + TT genotype carriers, the consumption of omega-3 fatty acids by the CC genotype carriers, supplying one percent of the energy requirement, reduced the risk of developing the MetS, high TG and low HDL-C up to 50%, 19%, and 32%, respectively. In addition, compared to the CT + TT genotype carriers, higher zinc consumption by the CC genotype carriers had reduced the risk of high FPG up to 50%.

## References

[1] Chimienti F (2006). *In vivo* expression and functional characterization of the zinc transporter ZnT8 in glucose-induced insulin secretion. J. Cell Sci..

[2] Lemaire K (2009). Insulin crystallization depends on zinc transporter ZnT8 expression, but is not required for normal glucose homeostasis in mice. Proc Natl Acad Sci USA.

[3] Hashemipour M (2009). Effect of zinc supplementation on insulin resistance and components of the metabolic syndrome in prepubertal obese children. Hormones (Athens, Greece).

[4] Cheng L, Zhang D, Zhou L, Zhao J, Chen B (2015). Association between SLC30A8 rs13266634 Polymorphism and Type 2 Diabetes Risk: A Meta-Analysis. Med Sci Monit..

[5] DeMenna J (2014). Association of common genetic variants with diabetes and metabolic syndrome related traits in the Arizona Insulin Resistance registry: a focus on Mexican American families in the Southwest. Hum Hered..

[6] Saxena R (2007). Genome-wide association analysis identifies loci for type 2 diabetes and triglyceride levels. Science.

[7] Kommoju UJ (2013). No detectable association of IGF2BP2 and SLC30A8 genes with type 2 diabetes in the population of Hyderabad, India. Meta gene..

[8] Imamura M, Maeda S (2011). Genetics of type 2 diabetes: the GWAS era and future perspectives [Review]. Endocr J..

[9] Kanoni S (2011). Total zinc intake may modify the glucose-raising effect of a zinc transporter (SLC30A8) variant: a 14-cohort meta-analysis. Diabetes..

[10] Hruby A (2013). Higher magnesium intake is associated with lower fasting glucose and insulin, with no evidence of interaction with select genetic loci, in a meta-analysis of 15 CHARGE Consortium Studies. J. Nutr..

[11] Azizi F (2002). Cardiovascular risk factors in an Iranian urban population: Tehran lipid and glucose study (phase 1). Soz Praventivmed..

[12] Farahmand M, Tehrani FR, Amiri P, Azizi F (2012). Barriers to healthy nutrition: perceptions and experiences of Iranian women. BMC public health..

[13] The Nutrient Data Laboratory. Food Composition Table (FCT), food and nutrition information center, United State Department of Agriculture (USDA). http://www.nal.usda.gov/fnic/foodcomp (2015) (Date of Access 7/1/2015).

[14] Azar, M. & Sarkisian, E. Food Composition Table of Iran. Tehran: National Nutrition and Food Research Institute. Shahid Beheshti University Press (1980).

[15] Esfahani FH, Asghari G, Mirmiran P, Azizi F (2010). Reproducibility and relative validity of food group intake in a food frequency questionnaire developed for the Tehran Lipid and Glucose Study. J. Epidemiol..

[16] Hosseini-Esfahani F, Djazaieri SA, Mirmiran P, Mehrabi Y, Azizi F (2012). Which food patterns are predictors of obesity in Tehranian adults?. J. Nutr Educ Behav..

[17] Mirmiran P, Esfahani FH, Mehrabi Y, Hedayati M, Azizi F (2010). Reliability and relative validity of an FFQ for nutrients in the Tehran lipid and glucose study. Public Health Nutr..

[18] Ainsworth BE (2000). Compendium of physical activities: an update of activity codes and MET intensities. Med Sci Sports Exerc..

[19] Kriska AM (1990). Development of questionnaire to examine relationship of physical activity and diabetes in Pima Indians. Diabetes Care..

[20] Momenan AA (2012). Reliability and validity of the Modifiable Activity Questionnaire (MAQ) in an Iranian urban adult population. Arch Iran Med..

[21] Truett, G. E. *et al*. Preparation of PCR-quality mouse genomic DNA with hot sodium hydroxide and tris (HotSHOT). BioTechniques. **29**(1), 52, 4 (2000). 10.2144/00291bm09 10907076

[22] National center for biotechnology information. www.ncbi.nlm.nih.gov/ (2015) (Date of Access 4/5/2015).

[23] Grundy SM (2004). Implications of recent clinical trials for the National Cholesterol Education Program Adult Treatment Panel III guidelines. Arterioscler Thromb Vasc Biol..

[24] Azizi F (2010). Appropriate waist circumference cut-off points among Iranian adults: the first report of the Iranian National Committee of Obesity. Arch Iran Med..

[25] Jie Li (2010). Identification of high-quality cancer prognostic markers and metastasis network modules. Nat commun..

[26] Hosseini-Esfahani F (2015). Dietary patterns interact with APOA1/APOC3 polymorphisms to alter the risk of the metabolic syndrome: the Tehran Lipid and Glucose Study. Br J Nutr..

[27] Ebbesson SO, Risica PM, Ebbesson LO, Kennish JM, Tejero ME (2005). Omega-3 fatty acids improve glucose tolerance and components of the metabolic syndrome in Alaskan Eskimos: the Alaska Siberia project. Int J. Circumpolar Health..

[28] Singer P, Wirth M, Berger I (1990). A possible contribution of decrease in free fatty acids to low serum triglyceride levels after diets supplemented with n-6 and n-3 polyunsaturated fatty acids. Atherosclerosis..

[29] Packard CJ, Munro A, Lorimer AR, Gotto AM, Shepherd J (1984). Metabolism of apolipoprotein B in large triglyceride-rich very low density lipoproteins of normal and hypertriglyceridemic subjects. The J. Clin Invest..

[30] Leyton J, Drury PJ, Crawford MA (1987). Differential oxidation of saturated and unsaturated fatty acids *in vivo* in the rat. Br J Nutr..

[31] Parra D (2008). A diet rich in long chain omega-3 fatty acids modulates satiety in overweight and obese volunteers during weight loss. Appetite..

[32] Lorente-Cebrian S, Bustos M, Marti A, Martinez JA, Moreno-Aliaga MJ (2009). Eicosapentaenoic acid stimulates AMP-activated protein kinase and increases visfatin secretion in cultured murine adipocytes. Clin Sci (Lond)..

[33] Molinar-Toribio E (2015). Effect of n-3 PUFA supplementation at different EPA:DHA ratios on the spontaneously hypertensive obese rat model of the metabolic syndrome. Br J. Nutr..

[34] Dodson G, Steiner D (1998). The role of assembly in insulin’s biosynthesis. Current opinion in structural biology. Curr Opin Struct Biol..

[35] Tang X, Shay NF (2001). Zinc has an insulin-like effect on glucose transport mediated by phosphoinositol-3-kinase and Akt in 3T3-L1 fibroblasts and adipocytes. J. Nutr..

[36] Prasad AS, Bao B, Beck FW, Kucuk O, Sarkar FH (2004). Antioxidant effect of zinc in humans. Free Radic Biol Med..

[37] Brand IA, Kleineke J (1996). Intracellular zinc movement and its effect on the carbohydrate metabolism of isolated rat hepatocytes. J. Biol Chem..

[38] Corella D, Ordovas JM (2012). Interactions between dietary n-3 fatty acids and genetic variants and risk of disease. Br J. Nutr..

[39] Tai ES (2005). Polyunsaturated fatty acids interact with the PPARA-L162V polymorphism to affect plasma triglyceride and apolipoprotein C-III concentrations in the Framingham Heart Study. J Nutr..

[40] Shan Z (2014). Interactions between zinc transporter-8 gene (SLC30A8) and plasma zinc concentrations for impaired glucose regulation and type 2 diabetes. Diabetes..

[41] Sun Q, van Dam RM, Willett WC, Hu FB (2009). Prospective study of zinc intake and risk of type 2 diabetes in women. Diabetes care..

[42] Ma Y, He FJ, MacGregor GA (2015). High salt intake: independent risk factor for obesity?. Hypertension..

[43] Doaei S, Gholamalizadeh M (2014). The association of genetic variations with sensitivity of blood pressure to dietary salt: A narrative literature review. ARYA atherosclerosis.

